# Commentary: Systemic lupus erythematosus and cardiovascular disease: A Mendelian randomization study

**DOI:** 10.3389/fimmu.2022.1075400

**Published:** 2022-12-08

**Authors:** Sophia Kerns, Katherine A. Owen, Jessica Kain, Peter E. Lipsky

**Affiliations:** ^1^ AMPEL BioSolutions, LLC, Charlottesville, VA, United States; ^2^ ReImagined Lupus Investigation, Treatment and Education (RILITE) Research Institute, Charlottesville, VA, United States

**Keywords:** Mendelian randomization, systemic lupus - erythematosus, cardiovascular disease (CVD), heart failure, GWAS - genome-wide association study

Systemic lupus erythematosus (SLE) is characterized by immune dysregulation and multi-organ inflammation that is frequently associated with the development of cardiovascular disease (1). Despite evidence from large observational studies linking the two diseases (2), a causal genetic relationship between SLE and CVD has never been established. Mendelian randomization (MR) is a causal inference method that measures and correlates the effect sizes of exposure-associated genetic variants in large-scale genetic association studies on traits of interest. We therefore read with great interest the recent report by Gao and colleagues (3) which used MR methods to explore the potential genetic link between lupus and a number of cardiovascular diseases.

One of the primary findings in Gao et al. is the MR analysis showing that genetic susceptibility to SLE is associated with a higher risk of heart failure (HF). There is, however, some ambiguity concerning the datasets used for analysis. The main text states that the outcome GWAS for HF is derived from FinnGen and appears to be “All-Cause Heart Failure” (finn-b-I9_HEARTFAILURE_ALLCAUSE) which includes 23,397/194,811 cases/controls. While this dataset is also listed by Gao et al. (3) as the HF outcome data source in their Table 1, it is mislabeled and actually refers to a large meta-analysis by Shah et al. (4) (ebi-a-GCST009541) which uses 47,309/930,014 cases/controls. Subsequent analyses using 2-sample MR result in significant positive causal estimates for SLE on the FinnGen HF GWAS using 6 different methods tested, including MR-RAPS, MR_PRESSO, MR-Egger, Weighted Median, IVW (fixed) and Maximum Likelihood. However, a re-evaluation of these data presented here in **Figure 1A**, using the published instrumental variables (IVs) from Bentham et al. (5) as the exposure and the FinnGen All cause HF *or* ebi-a-GCST009541 as the outcome, reveals largely disparate results. Using a number of MR methods including those evaluated in Gao et al., as well as other methods available *via* the MR-base platform (www.mrbase.org), we determined positive causal estimates for SLE on HF for 10/17 MR methods tested using the FinnGen-All cause HF GWAS, whereas all methods were insignificant using the dataset from Shah et al. (ebi-a-GCST009541). Additional analyses using finn-b-I9_HEARTFAIL_NS (not-strict) or finn-b-I9_HEARTFAIL (strict), that include a narrower range of endpoints for patient inclusion, as the outcome also show inconsistent results, with the broader HF determination resulting in positive causal estimates in 9/17 methods and the stricter definition revealing causality for only 4/17 methods. Inconsistencies across these datasets may point to the larger issue in defining HF, with some studies including any defect of cardiac pump function and those that are restricted to diagnoses based on compromised left ventricular ejection fraction. We recognize the challenges that often accompany analysis of heterogenous conditions such as heart failure or SLE and commend the authors for their attempts to increase our understanding of the complicated relationship between lupus and cardiovascular disease. Nonetheless, we feel that in this complex field, it is essential that precise identification of datasets be required and that, when possible, multiple datasets be employed to substantiate conclusions concerning possible causal relationships between clinical traits and/or diseases.

**Figure 1:**
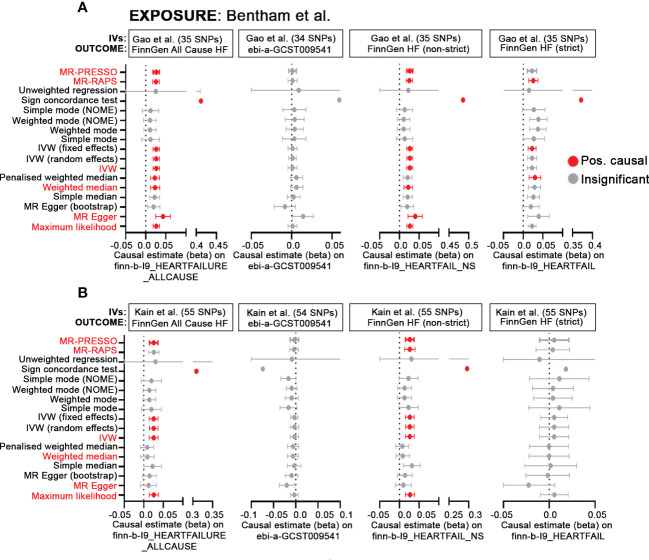
MR demonstrates inconsistent causal effects of SLE-associated SNPs on HF. **(A, B)** Forest plots of MR causal estimates (beta ± standard error) for SLE on All Cause HF (finn-b-I9_HEARTFAILURE_ALLCAUSE), ebi-a-GCST009541, finn-b-I9_HEARTFAIL_NS and finn-b-I9_HEARTFAIL. Summary statistics from the Bentham et al. GWAS (5) were used for the exposure. For results, grey indicates insignificant (p> 0.05), red, positive causal estimates (p<0.05) determined by each MR method. Red text indicates MR methods included in Gao et al. In **(A)**, IVs published in Gao et al. were derived solely from the Bentham et al. GWAS; for **(B)**, IVs were sourced from multiancestral datasets (5–7) and are published in Kain et al. (8).

A second issue in MR analysis relates to the limitations, correctly pointed out by the authors in their discussion, imposed by use of the comparatively smaller, European-only SLE GWAS (5) yielding limited power and genomic coverage. To maximize both power and scope, recent analyses completed by our group (8) have taken advantage of association studies using the Immunochip (6, 7), SLE GWAS (5) and variants pooled from the Phenoscanner database. Although still biased toward European ancestry, the combined use of multiancestral data may provide a more accurate picture of the genetic landscape underlying lupus and cardiovascular disease. Here, SLE IVs sourced from transancestral datasets along with the FinnGen All cause HF as the outcome reveals positive causal estimates for less than half (6/17) of the MR methods used (**Figure 1B**). MR-RAPS, MR-Egger and Weighted Median, which are 3 of the original methods (out of 6 total) used by Gao and colleagues, no longer maintain their significance. Inconsistent results are also observed using ebi-a-GCST009541(all insignificant), finn-b-I9HEARTFAIL_NS (not-strict; 7/17 positive causal) and finn-b-I9_HEARTFAIL (strict; all insignificant).

The totality of these results casts doubts on any conclusive causal relationship between genetic susceptibility of SLE and the risk of HF. They also highlight the need for larger, more inclusive SLE GWAS to test the hypothesis of causality between SLE and cardiovascular diseases. In an effort to maintain scientific transparency, we thank the editors of *Frontiers in Immunology* and the authors of the paper for recognizing our concerns.

## Author contributions

SK and JK wrote the first draft and contributed to data collection. KO and PL wrote and revised the final draft of the manuscript. All authors contributed to the article and approved the submitted version.
